# Combined Treatment of Uterine Leiomyosarcoma with Gamma Secretase Inhibitor MK-0752 and Chemotherapeutic Agents Decreases Cellular Invasion and Increases Apoptosis

**DOI:** 10.3390/cancers16122184

**Published:** 2024-06-10

**Authors:** Yasmin Abedin, Alexander Fife, Cherie-Ann Samuels, Rasheena Wright, Trystn Murphy, Xusheng Zhang, Emily Alpert, Emma Cheung, Qingshi Zhao, Mark H. Einstein, Nataki C. Douglas

**Affiliations:** 1 Department of Obstetrics, Gynecology, and Reproductive Health, Rutgers New Jersey Medical School, 185 S Orange Avenue, Newark, NJ 07103, USA; af1108@njms.rutgers.edu (A.F.); cns100@gsbs.rutgers.edu (C.-A.S.); rwright8@buffalo.edu (R.W.); tmm313@gsbs.rutgers.edu (T.M.); emily.alpert@pennmedicine.upenn.edu (E.A.); echeung@bowdoin.edu (E.C.); zhaoqi@njms.rutgers.edu (Q.Z.); me399@njms.rutgers.edu (M.H.E.); nd537@njms.rutgers.edu (N.C.D.); 2 Department of Genetics, Albert Einstein College of Medicine, 1301 Morris Park Avenue, Bronx, NY 10461, USA; xusheng.zhang@einsteinmed.edu; 3 Center for Immunity and Inflammation, Rutgers New Jersey Medical School, 185 S Orange Avenue, Newark, NJ 07103, USA

**Keywords:** uterine leiomyosarcoma, Notch signaling, gamma secretase inhibitors, chemotherapy, synergism, invasion, apoptosis, proliferation

## Abstract

**Simple Summary:**

Uterine leiomyosarcoma (uLMS) is a rare and aggressive malignancy with treatments that have limited efficacy and often have significant toxicities. We previously demonstrated the presence of Notch signaling in uLMS as well as decreased cell viability and Notch expression after exposure to the gamma secretase inhibitor MK-0752. The aim of this study was to assess the combination of MK-0752 and chemotherapeutics commonly used for the treatment of uLMS. Using two human uLMS cell lines, SK-UT-1B and SK-LMS-1, we demonstrated synergism between MK-0752 and docetaxel, doxorubicin, and gemcitabine using MTT assays. These synergistic combinations were used in transwell invasion assays, cell cycle flow cytometry, proliferation assays, and RNA sequencing. We found decreased invasion, an increase in the apoptotic sub-G1 cell cycle population, and differentially expressed genes and altered KEGG pathways after combination treatment. The combinations of MK-0752 and doxorubicin, MK-0752 and docetaxel, and MK-0752 with gemcitabine and docetaxel are potential novel therapeutic approaches for uLMS.

**Abstract:**

Due to limited effective therapeutics for uterine leiomyosarcoma (uLMS), the impact of the gamma secretase inhibitor (GSI) MK-0752 with common chemotherapeutics was explored in uLMS. MTT assays were performed on two human uLMS cell lines, SK-UT-1B and SK-LMS-1, using MK-0752, docetaxel, doxorubicin, and gemcitabine, individually and in combination, to determine cell viability after treatment. Synergistic combinations were used in transwell invasion assays, cell cycle flow cytometry, proliferation assays, and RNA sequencing. In SK-UT-1B, MK-0752 was synergistic with doxorubicin and gemcitabine plus docetaxel. In SK-LMS-1, MK-0752 was synergistic with all individual agents and with the combination of gemcitabine plus docetaxel. MK-0752, gemcitabine, and docetaxel decreased invasion in SK-UT-1B 2.1-fold* and in SK-LMS-1 1.7-fold*. In SK-LMS-1, invasion decreased 1.2-fold* after treatment with MK-0752 and docetaxel and 2.2-fold* after treatment with MK-0752 and doxorubicin. Cell cycle analysis demonstrated increases in the apoptotic sub-G1 population with MK-0752 alone in SK-UT-1B (1.4-fold*) and SK-LMS-1 (2.7-fold**), along with increases with all combinations in both cell lines. The combination treatments had limited effects on proliferation, while MK-0752 alone decreased proliferation in SK-LMS-1 (0.63-fold**). Both MK-0752 alone and in combination altered gene expression and KEGG pathways. In conclusion, the combinations of MK-0752 with either doxorubicin, docetaxel, or gemcitabine plus docetaxel are potential novel therapeutic approaches for uLMS. (* *p* < 0.05, ** *p* < 0.01).

## 1. Introduction

Uterine leiomyosarcoma (uLMS) is a rare and aggressive malignancy arising from smooth-muscle cells of the uterus with a five-year survival rate of less than 50% in individuals diagnosed in the early stages and an even lower five-year survival rate in those diagnosed with advanced-stage disease [1,2,3,4,5]. Characterized by its ability to mimic other benign gynecologic conditions and its potential for rapid growth and metastasis, uLMS remains a challenge to diagnose and treat [1,2,3]. 

The management of uLMS depends on the disease stage. Surgery is the primary treatment option for early-stage disease [2]. Adjuvant therapy in the setting of early-stage disease is not utilized, as it has not shown benefit compared to observation alone, and two-year recurrence rates remain high, between 50% and 70% [1,2,3]. Advanced-stage disease may incorporate surgery with adjuvant chemotherapy; however, there have not been any randomized studies directly comparing different treatment modalities in advanced disease. 

Doxorubicin, a potent anthracycline chemotherapeutic, is a standard first-line agent in the management of advanced uLMS [6,7,8]. Recently, the combination of doxorubicin and trabectedin was found to be efficacious against unresectable or metastatic LMS, including uLMS [9]. Despite the effectiveness of doxorubicin with or without trabectedin, these agents are associated with side effects such as cardiotoxicity and bone marrow suppression, which necessitate close monitoring and may limit treatment [3,6,9,10]. The combination of gemcitabine with docetaxel is another first-line therapy for advanced uLMS [6,7,8]. Gemcitabine inhibits DNA synthesis, impeding cell replication, while docetaxel interferes with microtubule function, disrupting the cell division process. When given in combination, gemcitabine and docetaxel are synergistic. Together, their efficacy against uLMS is better than either individual treatment. Clinical studies and trials have demonstrated encouraging results in terms of tumor response and progression-free survival rates in individuals receiving gemcitabine and docetaxel [3,11,12]. However, like many chemotherapy treatments that require multiple cycles, gemcitabine and docetaxel can cause adverse effects, including bone marrow suppression [3,6,11,12]. Due to the limited efficacy of the existing therapies for uLMS, it is imperative to identify new therapeutic approaches [4]. 

We previously investigated the involvement of the Notch signaling pathway, an evolutionarily conserved pathway with oncogenic roles in many cancers, in uLMS [13]. We determined Notch expression in uLMS tissue samples and uLMS cell lines, SK-UT-1B and SK-LMS-1. Compared to expression in benign tissues, uterine smooth muscle, and fibroids, we found increased expression of NOTCH3 and NOTCH4 in uLMS tissue samples. In the uLMS cells, the expression of NOTCH4 was higher in SK-LMS-1 compared to SK-UT-1B. Notch gene and protein expression were similar. *NOTCH1*, *NOTCH2*, and *NOTCH4*, but not *NOTCH3*, were detected in SK-LMS-1, and all four genes were expressed in SK-UT-1B. We then used gamma secretase inhibitors (GSIs) to indirectly inhibit Notch signaling in uLMS cells. The exposure of uLMS cells to GSIs, DAPT, and MK-0752, an orally well-tolerated GSI, decreased Notch signaling in a time- and dose-dependent manner, but did not impact the proliferation or invasion of SK-UT-1B and SK-LMS-1 cells [13]. Based on these findings, we concluded that inhibition of Notch signaling with MK-0752 has the potential to be effective against uLMS, but when used alone, the efficacy of MK-0752 would be minimal.

We sought to determine if MK-0752 in combination with common chemotherapeutics could be a novel therapeutic approach for uLMS. In both in vitro and in vivo ovarian cancer models, MK-0752 in combination with cisplatin decreased Notch signaling and inhibited cell growth [14]. When MK-0752 was used in combination with gemcitabine for pancreatic ductal adenocarcinoma, there was stable disease in 13 out of 19 evaluable patients [15]. In a phase I trial involving patients with breast cancer, increasing doses of MK-0752 with docetaxel was tolerable and there was a decrease in stem cell markers from biopsy specimens [16]. Based on these promising results and our prior findings, we hypothesized that combinations of MK-0752 with doxorubicin, gemcitabine, and/or docetaxel would be synergistic and would reduce tumor survival and disease progression. In this study, we first determined whether the combinations of MK-0752 and docetaxel, doxorubicin, or gemcitabine were synergistic, antagonistic, or additive against SK-UT-1B and SK-LMS-1 cells. Synergistic drug combinations were identified as those in which the total amount of two or more drugs needed to kill 50% of the cells was less than the sum of exact equal parts of each drug totaling one. To understand the impact of synergistic drug combinations on phenotypes associated with tumor progression and metastasis, we exposed uLMS cells to synergistic combinations that maintained cell viability at 70% or greater. We then assessed cellular invasion and proliferation and the proportion of uLMS cells in different phases of the cell cycle, including the apoptotic sub-G1 phase. Our data lay the foundation for developing in vivo models that investigate the efficacy of GSIs when used in combination with chemotherapy for treating uLMS.

## 2. Materials and Methods

### 2.1. Cell Lines and Cell Culture

Two human uLMS cell lines, SK-UT-1B (ATCC HTB-115™), a primary uLMS cell line with epithelial-like morphology, and SK-LMS-1 (ATCC HTB-88™), a vulvar metastasis from uLMS, were purchased from ATCC (Manassas, VA, USA) [13]. uLMS cell lines were cultured in Eagle’s Minimum Essential Medium (EMEM) (ATCC) with 10% fetal bovine serum (FBS) and 1% Pen-Strep. All cells were maintained in a humidified incubator under standard culture conditions of 21% O_2_ and 5% CO_2_ at 37 °C. SK-UT-1B and SK-LMS-1 cells were grown to greater than 80% confluence in 100 mm plates prior to conducting experiments. 

### 2.2. Inhibitory Concentrations of MK-0752, Docetaxel, Doxorubicin, and Gemcitabine

MK-0752 (MedChemExpress, Monmouth, NJ, USA), docetaxel (ab141248), doxorubicin (ab120629), and gemcitabine (ab145657) (Abcam, Cambridge, UK) were dissolved in dimethyl sulfoxide (DMSO) as the vehicle control. 

As previously described, MTT (3-(4,5-dimethylthiazol-2-yl)-2,5-diphenyltetrazolium bromide) assays (ab211091) (Abcam, Cambridge, UK) were used to determine cell viability after treatment of SK-UT-1B and SK-LMS-1 with MK-0752, docetaxel, doxorubicin, or gemcitabine individually at increasing concentrations [13]. Briefly, an MTT assay is a calorimetric assay that determines cell viability after increasing concentrations of a particular drug exposure [13]. Cells were plated in a 96-well plate and allowed to attach. Cells were then treated with media containing 10–1000 µM MK-0752, 0.10 to 10 µM docetaxel, 0.10 to 10 µM doxorubicin, or 0.10 to 150 µM gemcitabine for 72 hours (h). To determine if there was cell death from the vehicle control, SK-UT-1B and SK-LMS-1 cells were treated with DMSO in amounts equivalent to those used to resuspend MK-0752 or each chemotherapy. Cells were then incubated with 150 mL of freshly diluted 0.5 mg/mL MTT solution for 2–3 h at 37 °C. Thereafter, 150 mL of DMSO was added to each well, followed by incubation for 30 min at 37 °C, and absorbance was measured at 590 nm in a microtiter plate reader. Dose–response curves were generated and inhibitory concentrations (ICs) were determined for IC_30_ using the half maximal inhibitory concentration (IC_50_) and hillslope of the curves on GraphPad Prism Version 9.0 (San Diego, CA, USA). Samples were run in triplicate and experiments were repeated for at least n = 3; then, IC_50_ values were averaged for each agent.

After individual dose–response curves were generated, MK-0752 was combined with each chemotherapeutic agent that had reproducible results. The diagonal method was used to determine if combinations of MK-0752 and docetaxel, doxorubicin, or gemcitabine were more effective at decreasing uLMS cell viability than single agents [17]. When utilizing the diagonal method, drugs were prepared as a 1:1 mixture with a starting concentration 8 times the IC_50_ of each individual drug (8× IC_50_ drug A + 8× IC_50_ drug B). The treatments were then serially diluted, and a dose–response curve was generated by the MTT assay as described above. The IC_50_ of this curve was then used to determine if the relationship between the drugs was synergistic, antagonistic, or additive. For an additive relationship, half of the IC_50_ of drug A (0.5× IC_50_ drug A) in combination with half the IC_50_ of drug B (0.5× IC_50_ drug B) was the same as the IC_50_ of the combination therapy, and this was defined as 1X. A synergistic relationship was defined as an IC_50_ < 1X and an antagonistic relationship was defined as IC_50_ > 1X. Samples were run in triplicate and experiments were repeated for at least n = 3; then, the IC_50_ values were averaged for each combination. Cells were treated for 72 h and it was then determined whether there was greater efficacy using drug combinations as compared to individual agents [18]. Invasion assays were then performed on the cell lines with inhibitory concentrations of synergistic drug combinations at which 70% of SK-UT-1B and SK-LMS-1 cells were viable (IC_30_). The IC_30_ concentration was used with the intention of inducing moderate cytotoxicity while maintaining sufficient cell viability for analysis of cellular phenotypes. The IC_30_ of each drug in the combination was determined using the IC_50_ of the combination and was less than the IC_30_ concentration of each individual agent. The coefficient of the IC_50_ of the combination was divided by the number of agents in the combination (either 2 or 3). Then, this new value was multiplied by the original IC_50_ of the drug, yielding a new IC_50_. This value was then used along with the hillslope of the combination dose–response curve to yield the IC_30_ for each individual drug in the combination therapy. 

### 2.3. Transwell Invasion Assays 

**Figure 1:**
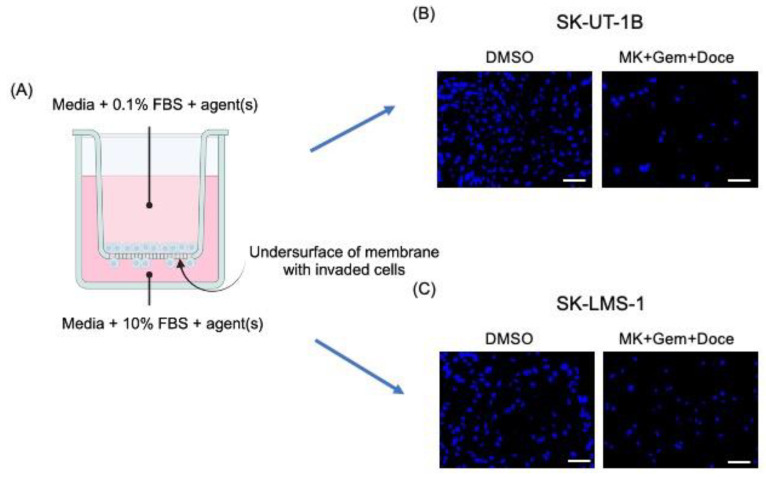
Schematic of the transwell invasion assay with representative images. (**A**) The schematic of the transwell invasion assay shows media with 0.1% FBS and agent(s) in the upper chamber and media with 10% FBS, as a chemoattractant, and agent(s) in the lower chamber. After 72 h of exposure to agent(s), cells on the undersurface of the membrane were fixed and stained with DAPI. Representative images of the membrane, after exposure to DMSO or MK-072 + gemcitabine + docetaxel, are shown for SK-UT-1B (**B**) and SK-LMS-1 (**C**). (**A**) was created with BioRender.com. (accessed on the 16th of May, 2024). MK = MK-0752, Gem = gemcitabine, Doce = docetaxel. Scale bars are 100 µm.

Invasion assays were performed in 24-well transwell chambers (Corning, Corning, NY, USA) as previously described [13]. Falcon transparent cell culture inserts (Corning, 8 µm pore size) were coated with growth-factor reduced, phenol red-free Matrigel^TM^ (Corning) mixed with media at a final concentration of 0.3 mg/mL (thick coating) and incubated for two hours at 37 °C. SK-UT-1B and SK-LMS-1 cells were serum-starved for 4 h before resuspension in serum-reduced media (0.1% FBS) at the IC_30_ concentration of MK-0752, each chemotherapeutic agent alone (docetaxel, doxorubicin, or gemcitabine), MK-0752 in combination with the chemotherapeutic agents, or DMSO, and added to the upper compartment. Chemoattractant medium (10% FBS) was added to the lower compartment with each respective agent to maintain a homogenous concentration of the agent(s) between chambers. After 72 h, cells on the undersurface of the membrane were fixed with 4% paraformaldehyde (PFA) and rinsed with phosphate-buffered saline (PBS). The membrane was excised with a scalpel, mounted on a glass slide with Vectashield containing 40, 6-diamidino-2-phenylindole (DAPI, Vector, Burlingame, CA, USA), and a coverslip was placed on top. Slides were imaged using the Keyence BZ-X710 All-in-One Fluorescent Microscope (Keyence, Osaka, Japan) at 10× magnification. Two independent observers counted the total number of cells on the undersurface of each membrane using ImageJ Fiji Version 2.1.0/1.53c [19]. Relative invasion for each treatment was represented as the number of cells present on the undersurface of the Matrigel layer normalized to those in the DMSO vehicle control. Additional comparisons between treatments were made. A schematic of the transwell invasion assay is demonstrated in Figure 1A, along with representative images of the DAPI-stained undersurface of the membrane for MK-0752 with gemcitabine and docetaxel compared to the DMSO control for SK-UT-1B (Figure 1B) and SK-LMS-1 (Figure 1C).

### 2.4. Cell Cycle Analysis

SK-UT-1B and SK-LMS-1 cells were serum-starved for 4 h and then treated with the DMSO vehicle control, IC_30_ concentration of MK-0752 alone, or MK-0752 in combination with chemotherapeutic agents that had decreased cellular invasion in the invasion assays. After 72 h, cells were harvested and fixed using 70% ethanol. For cell cycle analyses, fixed cells were stored at −20 °C for up to 2 weeks. Fixed cells were stained with a solution containing 50 μg/mL propidium iodide (Sigma Aldrich, St. Louis, MO, USA) and flow cytometry was performed [20]. After incubation at room temperature, cell cycle flow cytometry was performed on a BD LSR II flow cytometer. FloJo v10.9.0 was used for data analysis.

### 2.5. Cellular Proliferation

Using a similar protocol to the one used for cell cycle flow cytometry, a proliferation assay was also performed. Immediately after harvesting, anti-Hu Ki-67 antibody (0.06 μg/test) (RRID: AB_10687464) (Invitrogen, Carlsbad, CA, USA) was added to the cells. After incubation at room temperature for 30 min, Ki-67 flow cytometry was performed on a BD LSR II flow cytometer. FloJo v10.9.0 was used for data analysis.

### 2.6. Reverse Transcription—Quantitiative PCR

SK-UT-1B and SK-LMS-1 cells were serum-starved for 4 h and then treated with the DMSO vehicle control, IC_30_ concentration of MK-0752 alone, or MK-0752 in combination with gemcitabine and docetaxel. After 72 h, cells were harvested, and total RNA was isolated with the RNeasy Plus Mini Kit (Qiagen, Germantown, MD, USA). RNA was reverse-transcribed into cDNA using qScript cDNA Supermix (Quanta Bio, Beverly, MA, USA). After amplification, relative *HES1* expression was determined by qPCR with QuantiNova SYBR Green PCR Kit (Qiagen, Germantown, MD, USA) using gene-specific primers for *HES1* (Forward, 5′-AAAGATAGCTCGCGGCATTC-3′; Reverse, 5′-AGGTGCTTCACTGTCATTTCCA-3′) and 18s rRNA as the housekeeper (Forward, 5′CCGGGCTTCTATTTTGTTGGT-3′; Reverse, 5′-AGGTGCTTCACTGTCATTTCCA-3′). 

### 2.7. RNA Sequencing

Treatments that resulted in a decrease in *HES1* expression relative to DMSO vehicle control were selected for bulk RNA sequencing at Albert Einstein College of Medicine Epigenomics Shared Facility. Total RNAs were quantified by Qubit (Invitrogen, Carlsbad, CA, USA) and RNA quality was assessed by Fragment Analyzer (Advanced Analytical Technologies, Inc., Orangeburg, NY, USA). Purified total RNA was used to prepare libraries following the protocol using Qiaseq Stranded RNA Library Kit with UDI and QIAseq FastSelect -rRNA HMR Kit (Qiagen Inc., Germantown, MD, USA) for Illumina sequencing. Libraries were quality-controlled using fluorometric quantitation (Qubit; Invitrogen, Carlsbad, CA, USA), Agilent 2100 bioanalyzer (Agilent, Santa Clara, CA, USA), and QPCR (Roche Light Cycler, Roche Diagnostics, Basel, Switzerland). RNASeq libraries were multiplexed and sequenced as 1 × 75 bp single end on NEXTSEQ 500 (Illumina, San Diego, CA, USA) following standard protocols. 

The sequencing files of each sample in FASTQ format were trimmed for adaptors using trim galore v0.3.7. Trimmed fastq files were then aligned against human genome hg38 using STAR aligner v2.7.9a. Ribosomal RNA was detected and removed using SortMeRNA v4.3.4. Aligned sequencing data were then converted to gene count matrices using STAR. Differential analyses were performed using R package DESeq2 v1.42.0. Differentially expressed genes were identified as those with log2FC ≥ |1| and adjusted *p* value (*p* adj) < 0.05. Gene set enrichment analysis was conducted using R package fgsea v1.28.0 against KEGG database. Heatmaps were generated using R package heatmap v1.0.12.

### 2.8. Statistical Analysis

Medians were compared using the Mann–Whitney U test. Data are presented as medians with interquartile ranges (IQRs) and reported as fold changes with *p* values. Statistical analyses were performed using GraphPad Prism Version 9.0. Statistical significance was defined as *p* < 0.05.

## 3. Results

### 3.1. Combination of MK-0752 and Chemotherapeutics Decreases uLMS Cell Viability

**Figure 2:**
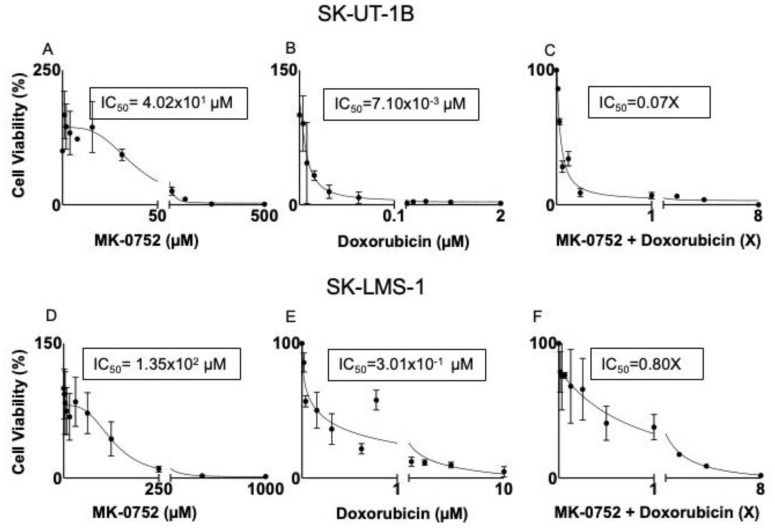
Representative dose–response curves for SK-UT-1B and SK-LMS-1. SK-UT-1B treated with MK-0752 with an IC50 of 4.02 × 10^1^ µM (**A**), doxorubicin with an IC50 of 7.10 × 10^−3^ (**B**), and the combination of the two agents with an IC50 of 0.07X (**C**). The combination of MK-0752 and doxorubicin is synergistic for SK-UT-1B. SK-LMS-1 treated with MK-0752 with an IC50 of 1.35 × 10^2^ µM (**D**), doxorubicin with an IC50 of 3.01 × 10^−1^ µM (**E**), and the combination of the two agents with an IC50 of 0.80X for 72 h (**F**). The combination of MK-0752 and doxorubicin is synergistic for SK-LMS-1.

After exposure of uLMS cell lines to individual or combination agents for 72 h, half-maximal inhibitory concentrations (IC_50_) were determined using an MTT assay. There was a decrease in cell viability in SK-UT-1B and SK-LMS-1 with increasing doses of all of the individual agents (Table 1) and combination agents (Table 2); however, not all combinations were synergistic. The IC_50_ values were then used with the hillslope of the curves to calculate the IC_30_ concentrations for each treatment condition. Representative dose–response curves identifying the IC_50_ are presented in Figure 2. Exposure of SK-UT-1B cells to the individual agents yielded an IC_50_ of 4.02 × 10^1^ µM and IC_30_ of 3.66 × 10^1^ µM for MK-0752 (Figure 2A, Table 1), an IC_50_ of 7.10 × 10^−3^ µM and IC_30_ of 3.70 × 10^−3^ µM for doxorubicin (Figure 2B, Table 1), an IC_50_ of 5.50 × 10^−5^ µM and IC_30_ of 2.40 × 10^−4^ µM for docetaxel (Table 1), and an IC_50_ of 2.00 × 10^−3^ µM and IC_30_ of 1.40 × 10^−3^ µM for gemcitabine (Table 1). 

Exposure of SK-LMS-1 cells to the individual agents yielded an IC_50_ of 1.35 × 10^2^ µM and IC_30_ of 8.12 × 10^1^ for MK-0752 (Figure 2D, Table 1), an IC_50_ of 3.01 × 10^−1^ µM and IC_30_ of 2.80 × 10^−1^ µM for doxorubicin (Figure 2E, Table 1), an IC_50_ of 1.00 × 10^−2^ µM and IC_30_ of 8.70 × 10^−3^ µM for docetaxel (Table 1), and an IC_50_ of 6.00 × 10^−2^ µM and IC_30_ of 2.00 × 10^−2^ µM for gemcitabine (Table 1). 

The diagonal method was then used to determine the relationship between MK-0752 and docetaxel, doxorubicin, or gemcitabine as synergistic, antagonistic, or additive for each cell line. Using this method, we determined the IC_50_ of the combination agents. For combinations with synergism, IC_30_ values were calculated and shown in Table 2. Exposure of SK-UT-1B to the combination of MK-0752 and gemcitabine yielded an IC_50_ of 1.05X, deeming it an antagonistic relationship (Table 2). In contrast, exposure of SK-UT-1B to MK-0752 and doxorubicin yielded an IC_50_ of 0.07X, deeming it a synergistic combination (Figure 2C, Table 2). The IC_30_ of each drug from this combination was 7.30 × 10^−1^ µM for MK-0752 and 1.00 × 10^−4^ µM for doxorubicin (Table 2). Exposure of SK-UT-1B cells to MK-0752, gemcitabine, and docetaxel yielded an IC_50_ of 0.70X, demonstrating synergism. The IC_30_ of each agent was 5.04 µM for MK-0752, 2.50 × 10^−5^ µM for gemcitabine, and 6.92 × 10^−5^ for docetaxel (Table 2). Exposure of SK-UT-1B to MK-0752 and docetaxel was not reproducible.

Exposure of SK-LMS-1 to MK-052 and docetaxel yielded an IC_50_ of 0.80X, deeming it a synergistic combination (Table 2). The IC_30_ of the individual drugs from this combination was 1.35 × 10^1^ µM for MK-0752 and 1.20 × 10^−3^ µM for docetaxel (Table 2). Exposure of SK-LMS-1 to MK-0752 and doxorubicin yielded an IC_50_ of 0.80X, which was another synergistic relationship (Figure 2F, Table 2). The IC_30_ of the individual drugs from the combination was 3.09 × 10^1^ µM for MK-0752 and 6.90 × 10^−2^ µM for doxorubicin (Table 2). Exposure of SK-LMS-1 to MK-0752 and gemcitabine yielded an IC50 of 0.30X, demonstrating synergism (Table 2). The IC_30_ of the individual drugs from this combination was 8.12 µM for MK-0752 and 3.50 × 10^−3^ µM for gemcitabine (Table 2). Since gemcitabine and docetaxel are used together in clinical practice, MK-0752 was combined with these two agents and this combination yielded an IC_50_ of 0.50X (Table 2). The IC_30_ of each agent from this regimen was 8.03 µM for MK-0752, 3.30 × 10^−4^ µM for gemcitabine, and 6.70 × 10^−4^ µM for docetaxel (Table 2). 

The IC_30_ concentrations of each agent from the synergistic combinations (Table 2) were lower doses than the IC_30_ concentrations for each individual agent (Table 1), and these lower doses were used for the invasion assays, cell cycle analyses, proliferation assays, and RNA sequencing.

### 3.2. Exposure to Combinations of MK-0752 and Chemotherapeutics Impacts uLMS Cellular Invasion

**Figure 3:**
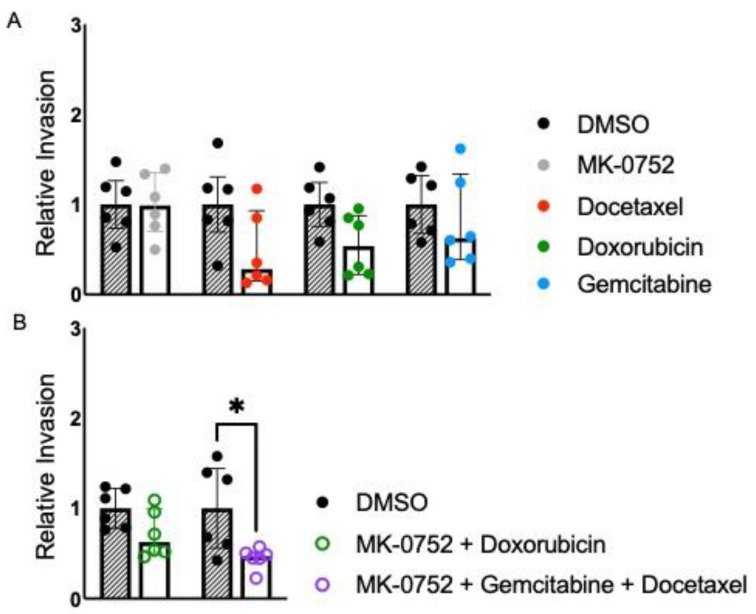
Relative invasion for SK-UT-1B exposed to single or combination agents for 72 h. (**A**). Cellular invasion was similar when SK-UT-1B cells were exposed to the IC30 concentration of MK-0752 (FC: docetaxel, doxorubicin, and gemcitabine, compared to the DMSO equivalent for 72 h). (**B**). Cellular invasion was decreased by 2.1-fold (*p* < 0.05) when cells were exposed to the IC30 of MK-0752, gemcitabine, and docetaxel compared to the equivalent DMSO control. Data are presented as median + IQR and * *p* < 0.05.

The invasion of cancer cells into surrounding tissues is one of the hallmarks of cancer metastasis. To determine if combinations of MK-0752 and chemotherapeutics decreased cellular invasion in uLMS cells, we assessed invasion after 72 h of exposure to individual or combination agents compared to the DMSO equivalent at the IC_30_ concentrations (Table 1 and Table 2). For SK-UT-1B, invasion was similar when SK-UT-1B cells were exposed to the IC_30_ concentration of MK-0752, docetaxel, doxorubicin, and gemcitabine, compared to the DMSO equivalent (Figure 3A). There was also no difference in cellular invasion when SK-UT-1B cells were exposed to the combination of MK-0752 and doxorubicin. In contrast, cellular invasion was decreased 2.1-fold (*p* < 0.05) when SK-UT-1B cells were exposed to the IC_30_ of MK-0752, gemcitabine and docetaxel compared to the equivalent DMSO control (Figure 3B). However, there was no significant difference in relative cellular invasion when single-agent treatments were compared to combination treatments in SK-UT-1B (Table 3).

**Figure 4:**
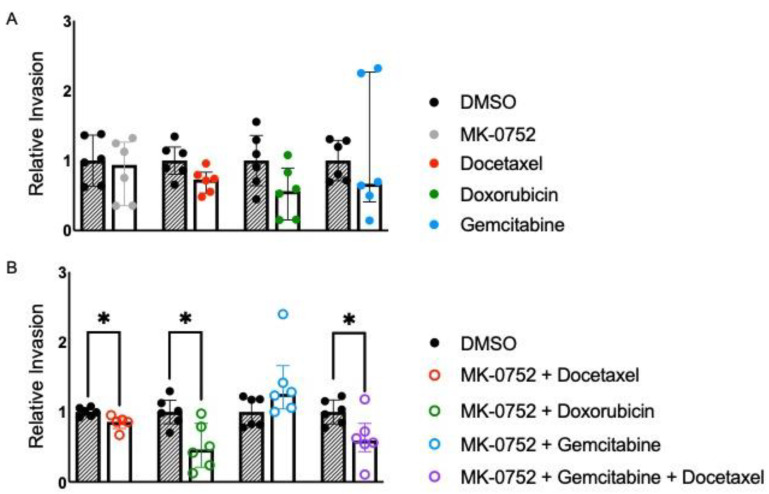
Relative invasion for SK-LMS-1 exposed to single or combination agents for 72 h. (**A**). Cellular invasion was similar when SK-LMS-1 cells were exposed to the IC30 concentration of MK-0752, docetaxel, doxorubicin, or gemcitabine, compared to the DMSO equivalent. (**B**). Cellular invasion was significantly decreased by 1.2-fold (*p* < 0.05) with exposure to the IC30 of MK-0752 and docetaxel, by 2.2-fold (*p* < 0.05) with exposure to MK-0752 and doxorubicin, and by 1.7-fold (*p* < 0.05) with exposure to MK-0752, gemcitabine, and docetaxel compared to the DMSO equivalent. Data are presented as medians + IQR and * *p* < 0.05.

For SK-LMS-1, invasion was similar when cells were exposed to the IC_30_ concentration of individual agents MK-0752, docetaxel, doxorubicin, and gemcitabine for 72 h compared to the DMSO equivalent (Figure 4A). Cellular invasion was also similar when SK-LMS-1 cells were exposed to the combination of MK-0752 and gemcitabine. Cellular invasion was significantly decreased 1.2-fold (*p* < 0.05) with exposure of SK-LMS-1 to the IC_30_ of MK-0752 and docetaxel, 2.2-fold (*p* < 0.05) with exposure to MK-0752 and doxorubicin, and 1.7-fold (*p* < 0.05) with exposure to MK-0752, gemcitabine, and docetaxel compared to the DMSO equivalent (Figure 4B). Further, there was a significant decrease in relative invasion (fold change: 0.47, *p* < 0.05) when SK-LMS-1 cells were treated with MK-0752, gemcitabine, and docetaxel, compared to the combination of MK-0752 with gemcitabine. However, there was no significant difference in relative invasion when the other single or combination treatments were compared to their respective combination treatments (Table 3).

### 3.3. Treatment of uLMS Cells with MK-0752 or MK-0752 in Combination with Chemotherapeutic Agents Affects the Cell Cycle 

**Figure 5:**
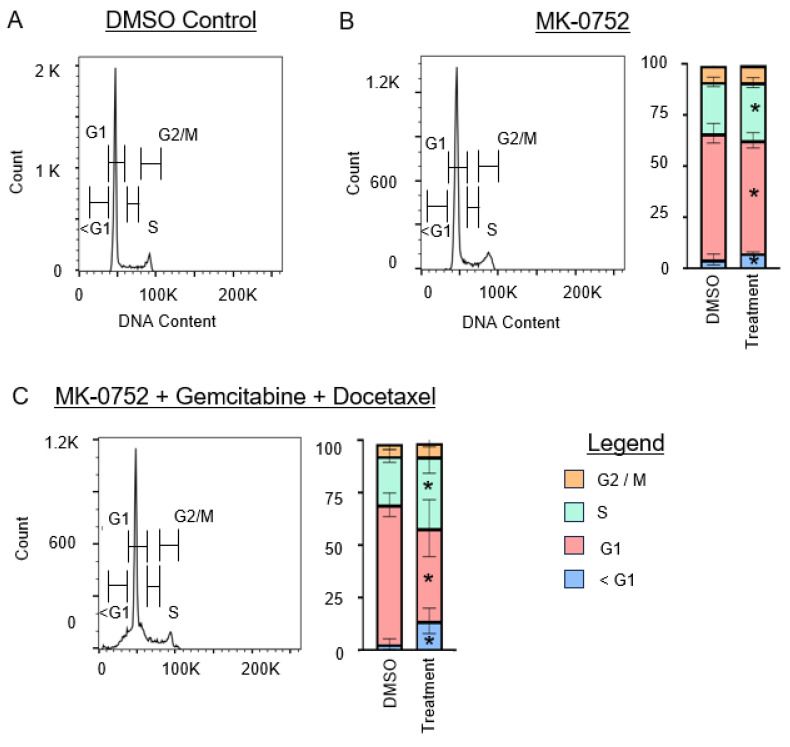
Cell cycle analysis for SK-UT-1B after exposure to single or combination agents for 72 h. (**A**). Representative image of the cell cycle flow cytometry curve for DMSO vehicle control. (**B**,**C**). Representative images of the cell cycle flow cytometry curve followed by stacked bar graphs to quantify the percentage of cells in each phase of the cell cycle after exposure to the IC30 concentration of MK-0752 alone (**B**) or in combination with gemcitabine and docetaxel (**C**) relative to equivalent volume of DMSO vehicle control. Data are presented as median + IQR and * *p* < 0.05.

**Figure 6:**
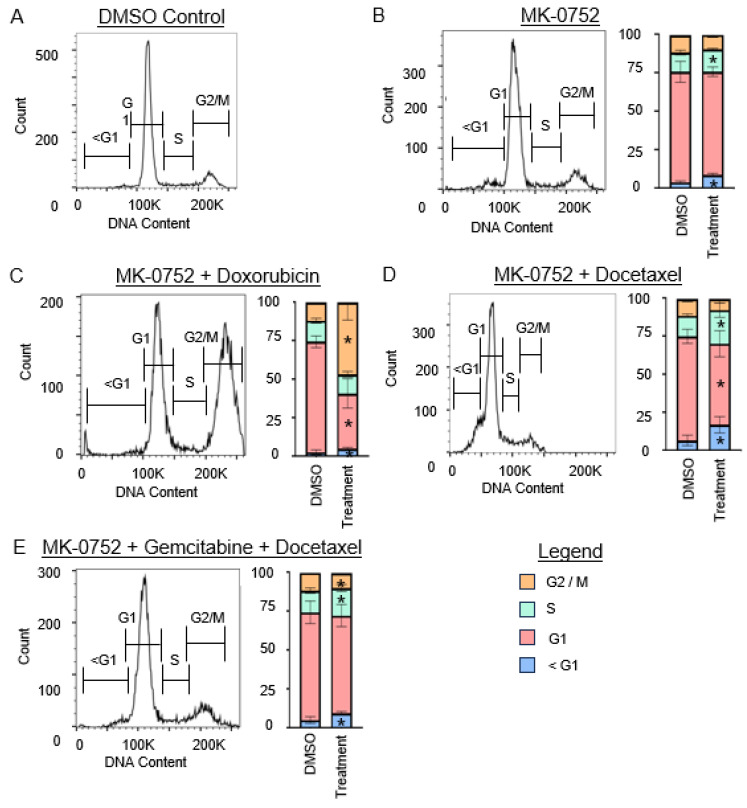
Cell cycle analysis for SK-LMS-1 cells after exposure to single or combination agents for 72 h. (**A**). Representative image of the cell cycle flow cytometry curve for DMSO vehicle control. (**B**–**E**). Representative images of the cell cycle flow cytometry curve followed by stacked bar graphs to quantify the percentage of cells in each phase of the cell cycle after exposure to the IC_30_ concentration of MK-0752 alone (**B**) or in combination with chemotherapeutics (**C**–**E**) relative to equivalent volume of DMSO vehicle control. Data are presented as median + IQR and * *p* < 0.05.

Using flow cytometry with PI detection, analysis of the cell cycle was performed after exposure of SK-UT-1B and SK-LMS-1 cells to the IC_30_ of MK-0752 alone, or the IC_30_ combinations of MK-0752 and chemotherapeutic agents that decreased cellular invasion, or the equivalent volume of DMSO vehicle control for 72 h (Figure 5 and Figure 6). To understand whether increased apoptosis could have contributed to the decreased cellular invasion we observed, we determined the percentage of cells in the sub-gap 1 (G1) population. 

For SK-UT-1B (Figure 5), treatment with MK-0752 alone increased the sub-G1 population by 1.4-fold (*p* < 0.01). Combination therapy with MK-0752, gemcitabine, and docetaxel also increased this population by 4.5-fold (*p* < 0.01). For SK-LMS-1 (Figure 6), treatment with MK-0752 alone increased the sub-G1 cell population by 2.7-fold (*p* < 0.01), MK-0752 and doxorubicin by 2.1-fold (*p* = 0.01), MK-0752 and docetaxel by 2.4-fold (*p* < 0.01), and MK-0752, gemcitabine, and docetaxel by 1.8-fold (*p* < 0.01). We found that all treatments increased the apoptotic sub-G1 cell population, suggesting that a proportion of the cells surviving after treatment are in the process of experiencing cell death. 

We then assessed whether exposure of uLMS cells to MK-0752 and/or chemotherapeutic agents impacted other phases of the cell cycle, G1, synthesis (S), and gap 2/mitosis (G2/M) phases. For SK-UT-1B (Figure 5), the percentage of cells in the G1 phase decreased when cells were treated with MK-0752 (1.2-fold, *p* < 0.01) or MK-0752, gemcitabine, and docetaxel (1.7-fold, *p* = 0.02). There was a modest increase in the percentage of SK-UT-1B cells in the S phase after treatment with MK-0752 (1.1-fold, *p* = 0.04) or with MK-0752, gemcitabine, and docetaxel (1.6-fold, *p* = 0.04). Compared to the vehicle control, there was no statistical difference in the percentage of SK-UT-1B cells in the G2/M phase after exposure to MK-0752 (*p* = 0.48) or the combination of MK-0752, gemcitabine, and docetaxel (*p* = 0.24).

For SK-LMS-1 (Figure 6), the percentage of cells in the G1 phase was decreased when cells were treated with MK-0752 and doxorubicin (1.7-fold, *p* < 0.01) or MK-0752 and docetaxel (1.3-fold, *p* < 0.01), but were unchanged when treated with MK-0752 alone (*p* = 0.07) or the combination of MK-0752, gemcitabine, and docetaxel (*p* = 0.48). The percentage of cells in the S phase increased after treatment of SK-LMS-1 cells with MK-0752 (1.2-fold, *p* = 0.04), MK-0752 and docetaxel (1.5-fold, *p* = 0.04), or MK-0752, gemcitabine, and docetaxel (1.3-fold, *p* = 0.02), but were unchanged after treatment with MK-0752 and doxorubicin (*p* = 0.26). Treatment of SK-LMS-1 cells with MK-0752 and doxorubicin increased the number of cells in the G2/M phase by 3.8-fold (*p* < 0.01), whereas treatment with MK-0752, gemcitabine, and docetaxel decreased the percentage of cells in G2/M by 1.2-fold (*p* = 0.02) and treatment with MK-0752 alone (*p* = 0.10) or MK-0752 and docetaxel (*p* = 1.0) had no impact on G2/M. 

### 3.4. Treatment of uLMS Cells with MK-0752 Decreases Proliferation Only in the SK-LMS-1 Cell Line

To assess changes in proliferation in surviving uLMS cells after exposure of cells to the IC_30_ of MK-0752 and chemotherapeutic combinations that were synergistic and decreased cellular invasion, Ki-67 expression was analyzed using flow cytometry. SK-UT-1B and SK-LMS-1 cells were exposed to the IC_30_ of MK-0752 alone, MK-0752 + doxorubicin, MK-0752 + docetaxel, MK-0752 + gemcitabine + docetaxel, and the equivalent volume of DMSO for 72 h. There were no changes in proliferation after any treatment in the SK-UT-1B cell line (Table 4). In the SK-LMS-1 cell line, there was a significant decrease in proliferation with exposure to MK-0752 alone (fold change 0.63, *p* < 0.01, Table 4).

### 3.5. Treatment with MK-0752 Alone and in Combination with Chemotherapeutics Yields Significant Changes in Differentially Expressed Genes

To determine global changes in gene expression, we performed bulk RNA sequencing on SK-UT-1B cells treated with the combination of MK-0752 with gemcitabine and docetaxel as well as SK-LMS-1 cells treated with MK-0752 alone and in combination with gemcitabine and docetaxel. These respective treatments for SK-UT-1B and SK-LMS-1 were chosen for RNA sequencing because they showed a decrease in HES1 expression by qPCR. The treatment of SK-UT-1B with MK-0752 alone was excluded due to no change in HES1 expression. Gene expression changes were observed in all treatments (Figure 8D–F). Treatment with the combination of MK-0752, gemcitabine, and docetaxel relative to DMSO elicited 287 up- and 172 downregulated differentially expressed genes in SK-UT-1B cells (*p* adj < 0.05 and log2FC ≥ |1|). Treatment of SK-LMS-1 cells with MK-0752 resulted in the significant upregulation of 62 and downregulation of 115 differentially expressed genes (*p* adj < 0.05 and log2FC ≥ |1|) and treatment with the combination of MK-0752, gemcitabine, and docetaxel resulted in 47 up- and 12 downregulated differentially expressed genes (*p* adj < 0.05 and log2FC ≥ |1|). Thus, the greatest changes in gene expression occurred in SK-UT-1B cells after exposure to MK-0752, gemcitabine, and docetaxel.

### 3.6. Cytotoxic Treatment Has Limited Effects on the Differences in Gene Expression and Altered Pathways in uLMS Cells between SK-LMS-1 and SK-UT-1B

**Figure 7:**
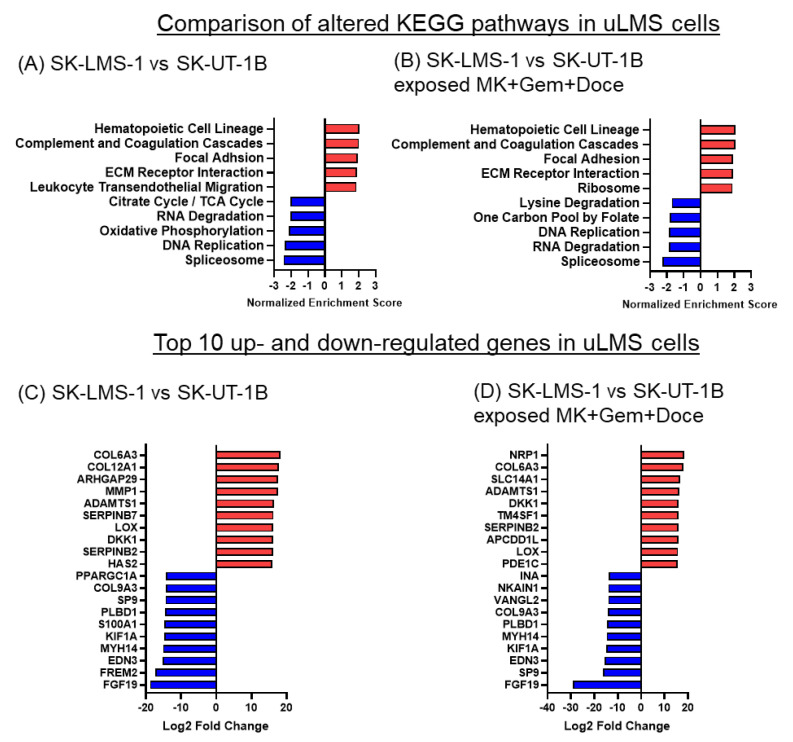
KEGG pathways and differentially expressed genes in SK-LMS-1 vs. SK-UT-1B cells before and after exposure to MK-0752 + gemcitabine + docetaxel. (**A**,**B**). The top 5 up (red) and downregulated (blue) KEGG pathways based on normalized enrichment scores and (**C**,**D**) the top 10 up- and downregulated significantly differentially expressed genes by log2 fold change after exposure to (**A**,**C**) DMSO vehicle control or (**B**,**D**) the combination of MK-0752, gemcitabine, and docetaxel at the IC_30_ for 72 h are depicted.

To determine global changes in gene expression and identify altered pathways in uLMS cells, we performed bulk RNA sequencing on SK-UT-1B cells and SK-LMS-1 cells after exposure to the DMSO vehicle control or combination of MK-0752, gemcitabine, and docetaxel. The hematopoietic cell lineage, complement and coagulation cascade, focal adhesion, and ECM receptor interaction pathways had higher expression in SK-LMS-1 relative to SK-UT-1B cells both after exposure to DMSO control and combination therapy (Figure 7A,B). The spliceosome and DNA replication pathways had lower expression in SK-LMS-1 relative to SK-UT-1B regardless of exposure (Figure 7A,B). When looking at gene expression, 13 of the 20 most differentially expressed genes between the two cell lines were the same whether exposed to DMSO or the combination treatment (Figure 7C,D). There are differences in multiple collagen genes (*COL6A3*, *COL9A3*, *COL12A1*), motor protein genes (*KIF1A*, *MYH14*), matrix metalloproteinase genes (*VANGL2*, *ADAMTS1*, *MMP1*), and angiogenesis genes (*EDN3*, *NRP1*), suggesting that many of the differentially expressed genes between the two uLMS cell lines are related to the morphologic differences between them.

### 3.7. MK-0752 Exposure Decreases Cell Cycle and DNA Replication Pathway Activity

**Figure 8:**
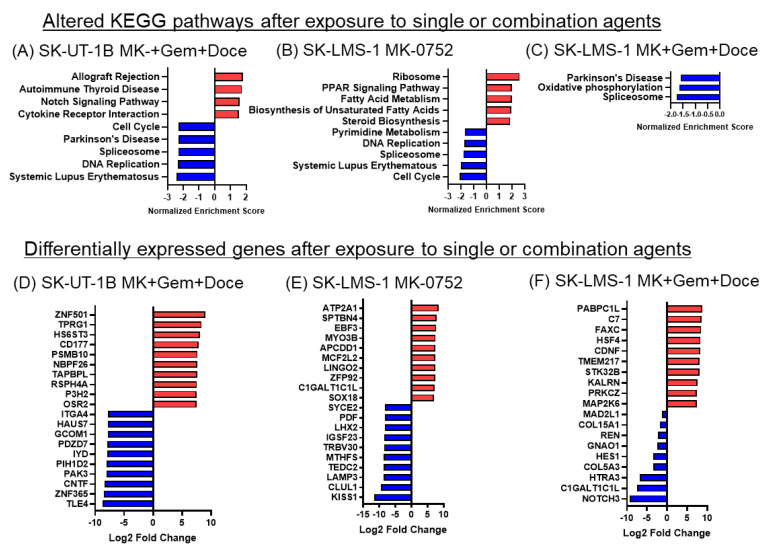
KEGG pathways and differentially expressed genes in uLMS cells exposed to single or combination agents. (**A**–**C**). The top 5 altered pathways are depicted unless fewer than 5 pathways met statistical significance as defined by *p* adj < 0.05, in which case only those pathways that were significantly altered are shown for (**A**) SK-UT-1B cells with MK-0752, gemcitabine, and docetaxel; (**B**) SK-LMS-1 with MK-0752; (**C**) SK-LMS-1 with MK-0752, gemcitabine, and docetaxel relative to DMSO vehicle control. (**D**–**F**). The top ten up (red) and downregulated (blue) significantly differentially expressed genes by log2 fold change after treatment of (**D**) SK-UT-1B cells with MK-0752, gemcitabine, and docetaxel; (**E**) SK-LMS-1 with MK-0752; (**F**) SK-LMS-1 with MK-0752, gemcitabine, and docetaxel relative to DMSO vehicle control.

Pathway analysis was performed on SK-UT-1B cells treated with the combination of MK-0752 with gemcitabine and docetaxel (Figure 8A) as well as SK-LMS-1 cells treated with MK-0752 alone (Figure 8B) and in combination with gemcitabine and docetaxel (Figure 8C). Treatment of SK-UT-1B cells with MK-0752, gemcitabine, and docetaxel increased the KEGG Notch signaling pathway and immune-related pathways and decreased DNA replication and cell cycle pathways (Figure 8A). Both SK-UT-1B and SK-LMS-1 cells had decreases in the Parkinson’s disease and spliceosome pathways after exposure to combination therapy (Figure 8A,C). Treatment of SK-LMS-1 with MK-0752 alone showed increases in fatty acid and steroid metabolism pathways as well as ribosome pathways in SK-LMS-1 (Figure 8B). Further, there were decreases in cell cycle, nucleotide metabolism and DNA replication pathways in SK-LMS-1 (Figure 8B). Treatment with the combination of MK-0752, gemcitabine, and docetaxel demonstrated no significant upregulation of any KEGG pathways in SK-LMS-1. The spliceosome pathway was decreased in all treatment groups. 

### 3.8. Expression of Notch Pathway Effectors Is Decreased and Expression of Components of the Gamma Secretase Complex Are Increased after Exposure to the Combination of MK-0752 and Chemotherapeutic Agents

**Figure 9:**
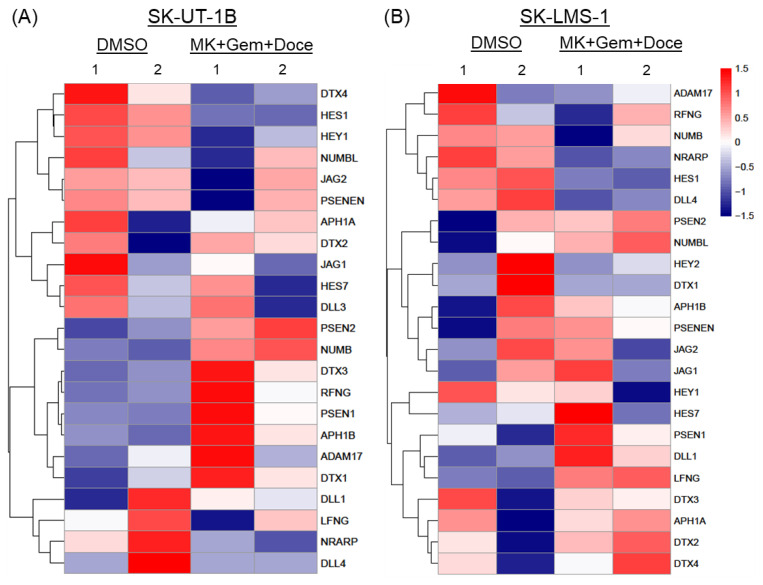
Heatmaps of differentially expressed genes in the Notch signaling pathway. Expression of 23 genes relating to ligands, regulators, and downstream effectors of the Notch signaling pathway after exposure to the combination of MK-0752, gemcitabine, and docetaxel at the IC_30_ for 72 h relative to DMSO vehicle control was compared in (**A**) SK-UT-1B and (**B**) SK-LMS-1 cells.

RNA seq analysis shows differential expression of 23 Notch pathway genes after the treatment of both SK-UT-1B and SK-LMS-1 cells with MK-0752, gemcitabine, and docetaxel relative to the DMSO vehicle control. There were similar results in both cell lines, with decreased expression of downstream effectors of the Notch signaling pathway, *HES1*, *NRARP*, and *HEY1*, confirming decreased Notch pathway activity after treatment (Figure 9). Genes encoding proteins that make up the gamma secretase complex (*APH1*, *PSEN1*, *PSEN2*, *PSENEN*) were upregulated. Additional upregulated genes included modulators of Notch signaling, including *DTX*, *NUMB,* and *RFNG*. The effects on genes encoding Notch ligand genes *DLL1-4* and *JAG1-2* were varied (Figure 9).

## 4. Discussion

Research on novel therapy regimens for uLMS is challenging due to the rarity of the disease; nonetheless, the poor prognosis associated with uLMS highlights the importance of such studies. Given that MK-0752 was well tolerated in phase I clinical trials for several malignancies and we previously showed that MK-0752 decreased the expression of *HES1* and decreased uLMS cell viability in a dose- and time-dependent manner in uLMS cell lines, we sought to explore the efficacy of MK-0752 in combination with doxorubicin, gemcitabine, and/or docetaxel in uLMS [13,15,16,21,22]. We first established the half maximal inhibitory concentrations of individual agents (MK-0752, docetaxel, doxorubicin, and gemcitabine) and then identified the relationship between the treatments. All combinations of MK-0752 with chemotherapeutics decreased cell viability with increasing doses, but not all combinations were synergistic. We then performed experiments to determine how synergistic drug combinations impacted SK-UT-1B and SK-LMS-1 cellular invasion, apoptosis, and proliferation, which are varying hallmarks of cancer growth and metastasis. When uLMS cells were exposed to single agents, there was no change in invasion compared to the vehicle control; however, there was a significant increase in apoptosis across both cell lines and a decrease in proliferation in SK-LMS-1. When uLMS cells were exposed to synergistic treatments, there was a significant decrease in invasion and an increase in apoptosis. KEGG pathway enrichment analysis revealed a decrease in cell cycle, spliceosome, and DNA replication pathways with exposure of both SK-UT-1B and SK-LMS-1 to synergistic treatments. Synergism enables the utilization of lower and better-tolerated doses of each agent, aiming to minimize the adverse effects of systemic therapy. Thus, these findings suggest that the addition of MK-0752 to standard chemotherapeutics for uLMS used in clinical practice may be more efficacious than chemotherapy alone. 

The relationship between MK-0752 and each chemotherapeutic agent was unique to the uLMS cell type. In SK-UT-1B, a primary uLMS cell line with epithelial morphology, only the combinations of MK-0752 + doxorubicin and MK-0752 + gemcitabine + docetaxel were found to be synergistic. When treatments were compared to one another, there was no significant change in invasion in SK-UT-1B. In SK-LMS-1, all four combinations, MK-0752 + docetaxel, MK-0752 + doxorubicin, MK-0752 + gemcitabine, and MK-0752 + gemcitabine + docetaxel, were found to be synergistic. Further, there was a decrease in relative invasion with MK-0752 + gemcitabine + docetaxel compared to MK + gemcitabine in SK-LMS-1. Although SK-UT-1B is not directly derived from an epithelioid sub-type of uLMS, which is known to be more aggressive than the conventional type, the epithelial morphology may reflect an aggressive phenotype and explain the lack of response to some combinations of drugs [6,13,23,24]. RNA sequencing data also showed that the SK-UT-1B cell line had increased activity in DNA replication- and metabolism-related pathways relative to SK-LMS-1. Baseline differences in gene expression between the two cell lines may contribute to the drug resistance seen in SK-UT-1B cells. Conversely, the lack of differences in invasion between each individual and combination treatment in SK-UT-1B and SK-LMS-1 could be attributed to limitations in our study design; specifically, the IC_30_ for individual and combination drug exposures was the only concentration we tested. Nevertheless, the synergy observed with MK-0752 + gemcitabine + docetaxel resulted in reduced invasion compared to the vehicle control for both SK-UT-1B and SK-LMS-1 cells, making this regimen a priority for further investigation.

We used RNA sequencing to gain a better understanding of how the combination of MK-0752 + gemcitabine + docetaxel impacts multiple genes associated with the Notch signaling pathway. Downstream effectors of the Notch signaling pathway, *HES1*, *NRARP*, and *HEY1*, were downregulated after treatment in both uLMS cell lines, confirming the established role of a GSI in Notch inhibition [13]. We observed upregulation of genes that encode components of the gamma secretase complex as well as modulators of the Notch signaling pathway, *DTX*, *RFNG*, and *NUMB*. In the DTX family (Deltex; family *DTX*), E3 ubiquitin ligase mediates Notch signaling [25] and can activate the pathway in a ligand-independent manner [26]. *RFNG* increases the activation of NOTCH1 by its ligands, DLL1 and JAG1 [27], and NUMB also has been shown to increase Notch signaling [28]. These data suggest that, in response to GSI and Notch inhibition, the expression of genes that would enhance activity of the Notch pathway or increase the presence of the gamma secretase enzyme is increased.

Notch inhibition with the GSI MK0752 increased the apoptotic sub-G1 cell population in both SK-UT-1B and SK-LMS-1 cell lines, both alone and in combination with common chemotherapeutic agents, indicating that a significant proportion of the cells surviving after treatment are in the process of undergoing cell death. There was no difference in the proportion of sub-G1 cells between MK-0752 alone or in combination with chemotherapeutics, suggesting MK-0752 was the primary contributor to the increased apoptosis. In other cancers, Notch inhibition with GSIs has similar increases in apoptosis; however, these increases have generally been accompanied by either a G1 phase arrest [29,30,31] or G2/M arrest [14,32]. For SK-UT-1B and SK-LMS-1 cells, we did not observe G1 phase arrest after any treatment. We found decreases in the G1 population and increases in the proportion of cells in the S phase. The total G1 and sub-G1 populations were similar between the treatment and control groups, suggesting that the apoptotic population may be originating from cells in the G1 phase. With MK-0752 and doxorubicin, there was a large increase in the percentage of cells in the G2/M phase of the cell cycle suggestive of a G2/M arrest. Prior research using doxorubicin in combination with roscovitine in SK-LMS-1 cells has also demonstrated a G2/M arrest, as have studies looking at doxorubicin in lymphoid and kidney tissue [20,33,34,35]. Our RNA sequencing data support the findings we saw in cell cycle flow cytometry. DNA replication and cell cycle pathways were downregulated in both cell lines. The Notch signaling pathway has an established role in proliferative signaling and the progression of many cancers [36]. Pairing the known role of the Notch pathway in proliferation and the effects on the cell after treatment suggests the mechanism of MK-0752 and Notch pathway inhibition affects its role in the downregulation of these pathways.

While we found a limited effect on proliferation after cell lines were exposed to combination treatments, there was a significant decrease in cellular proliferation after treatment with MK-0752 alone in the SK-LMS-1 cell line. We had previously not seen a change in proliferation in SK-LMS-1 after treatment with DAPT or MK-0752 at the IC_5_ for 24 h, so this decrease after treatment with the IC_30_ of MK-0752 over 72 h supports a dose- and time-dependent effect on proliferation [13]. Beyond our study, there has been limited research into the effects of MK-0752 on cellular proliferation to help aid interpretation of these results. MK-0752 in combination with endocrine therapy decreased *Ki-67* mRNA expression in estrogen receptor positive breast cancer [37]. An alternative GSI, RO4929097, in combination with temozolomide and radiotherapy, decreased proliferation in glioblastoma [38], whereas the GSI MRK-003 decreased proliferation in pancreatic ductal adenocarcinoma [39]. These studies, along with the decreased proliferation that we observed in our study, suggests an additional potential mechanism of MK-0752. Further, the change in invasion seen in SK-UT-1B and SK-LMS-1 with combination treatments is unlikely to be due to any changes in proliferation, but rather due to either an increase in apoptosis or an inherent decrease in the invasive nature of the cancer cell lines. 

RNA sequencing data suggested another potential role of gamma secretase inhibitors in the treatment of uterine leiomyosarcoma. Treatment with MK-0752 alone and in combination in both cell lines lead to a downregulation of the spliceosome, which is being investigated as a novel therapeutic target. The dysregulation of splicing is common in many cancers, and these mutations and the aberrant expression of spliceosome components can lead to enhanced tumor cell proliferation, invasion, metastasis, chemoresistance, and inhibition of apoptosis [40]. A broad range of spliceosome-targeted therapies (STT) and spliceosome inhibitors that affect the pathway at different points are under investigation [41]. The role of GSI’s and Notch inhibition on the spliceosome have not yet been investigated; however, this potential connection is one that may warrant further investigation.

There are few preclinical studies that have examined novel pathway targets in combination with common chemotherapeutics in LMS and uLMS in particular. Babichev et al. examined the role of phosphatidylinositol 3-kinase/AKT/mammalian target of the rapamycin (PI3K/AKT/mTOR) pathway inhibitors in conjunction with doxorubicin and found a decrease in cell viability with combination therapies in a soft tissue sarcoma cell line and in the SK-LMS-1 cell line. They also found that synergistic combinations reduced tumor volumes in the SK-LMS-1 xenograft model [18]. Lopez et al. combined the histone deacetylase (HDAC) inhibitor mocetinostat with gemcitabine in LMS cell lines, including SK-LMS-1. They found that their combination decreased cell growth in vitro and tumor size in vivo using a SK-LMS-1 derived xenograft model [42]. Although these studies only explored one combination therapy, they are, to our knowledge, the only existing reports exploring novel combination therapies for LMS. Further, clinical trials involving PI3K/AKT/mTOR or HDAC inhibitors in combination with chemotherapeutics are limited with minimal response in sarcomas. The phase II study of mocetinostat and gemcitabine in LMS included 8(40%) uLMS participants and the median time to disease progression was two months [43]. Therefore, more studies involving novel therapeutic strategies are needed, including the use of GSIs. 

To the best of our knowledge, our research is the first preclinical study to combine GSIs with common chemotherapeutics for uLMS with promising results and we would like to highlight several strengths. Herein, we evaluated two uLMS cell lines that are not only morphologically different, but also derived from distinct sites: SK-UT-1B is a uLMS cell line originating from the uterus, whereas SK-LMS-1 is from a vulvar metastasis of uLMS. This gives us insight into the variability and also similarity in cellular invasion in response to different drug exposures. We also explored more than one combination, which allowed us to determine a common and potentially optimal regimen for two different uLMS cell lines; the use of MK-0752 along with gemcitabine and docetaxel demonstrated a decrease in cellular invasion in both cell lines. Another strength of this study was the methodical approach of using the MTT assay with the diagonal method in order to explore the relationship between two or more drugs [17]. This has not been previously achieved using the drug combinations or cell lines in our study. Additionally, the concentrations of drugs used in our cell culture models were comparable to those in mouse and human studies, indicating that using scalable doses in mouse and human trials may yield potential similarity in cellular responses while minimizing toxicity [16,18,42,44,45]. We will use our comprehensive data for validation in further in vivo studies, paving the way for clinical trials.

Our study is limited by the general conditions of in vitro models, which restricts our understanding of the effect of these drugs on uLMS in vivo. Preclinical in vivo and clinical studies of doxorubicin and gemcitabine with docetaxel in uterine sarcomas, including uLMS, have shown moderate tumor response and increased progression-free survival [10,11,42]. We saw no change in cellular invasion in vitro, but we did see a decrease in cell viability and increase in apoptosis when our cell lines were exposed to these agents. Hence, experiments and outcomes explored in in vitro studies may be different than those in in vivo or human trials, and cellular invasion is not the only cell function that contributes to tumor progression. Further, the rarity of uLMS and sarcomas makes them difficult to study, as they are often diagnosed at the time of surgery or in the final pathology report. Diagnosing uLMS before surgery is uncommon. Consequently, studies involving fresh uLMS tissue or patient-derived xenograft (PDX) mouse models are scarce, while studies involving in vitro and cell line derived models are more common [13]. Some studies have used SK-LMS-1 as a cell-line derived xenograft model in mice and exploring this model will be the next step in our research [18,42]. We plan to compare the effects of treatment with MK-0752 alone to the combination of gemcitabine and docetaxel with and without MK-0752. The combination of MK-0752, gemcitabine, and docetaxel was synergistic, decreased invasion, and had cell cycle effects on both cell lines. Another limitation is that our experiments were performed only utilizing concurrent drug regimens for 72 h of exposure. Changing the chronicity and duration of exposure to agents may aid us in establishing a more appropriate regimen, given that MK-0752 is an oral agent that has previously been given weekly in phase I trials with a half-life of 15 h [15,18,22]. Moreover, only the effect of the IC_30_ concentration of drugs was studied, such that cells would be exposed to enough drug to exhibit changes in functionality, but not enough drug to cause 100% cell death. Utilizing other concentrations can help us determine an ideal dose and schedule. 

Our prior and current findings will help guide future directions. We previously found that expression of NOTCH3 and NOTCH4 was higher in uLMS samples than in benign uterine smooth muscle and fibroids [13]. Expression of NOTCH4 was also higher in SK-LMS-1 compared to SK-UT-1B [13]. Similar to uLMS, NOTCH3 and NOTCH4 have been implicated in various cancers. Exploring Notch-specific targets such as NOTCH3-targeted antibody drug conjugates and/or NOTCH4 monoclonal antibodies can help determine if there is greater tumor response and less toxicity with more specific targeted therapy in uLMS [46,47,48]. Further, comparing these agents to GSIs individually and in combination with chemotherapeutics with three-dimensional cell culture and in vivo models will aid in identifying the most effective treatments for uLMS. 

There remains a clinical and critical need for evaluating novel therapeutic strategies for uLMS. There are few clinical trials involving targeted therapies, including immunotherapy, tyrosine kinase inhibitors, hormone therapy, and DNA repair modulators such as poly-ADP ribose polymerase (PARP) inhibitors, combined with common chemotherapeutics [49]. Appreciating the limitations of in vitro models, we have identified the combinations of MK-0752 and doxorubicin, MK-0752 and docetaxel, and MK-0752 with gemcitabine and docetaxel as novel therapeutic approaches for uLMS. In vivo models and clinical trials will only help us determine the true efficacy of these drug regimens in uLMS. 

## 5. Conclusions

In conclusion, when uLMS cell lines SK-UT-1B and SK-LMS-1 were exposed to synergistic combination treatments, there was a significant decrease in invasion and increase in apoptosis. However, when these cell lines were exposed to single agents, there was no change in invasion, but there was still a significant increase in apoptosis across both cell lines and a decrease in proliferation in SK-LMS-1 with many differentially expressed genes and altered pathways. Future directions will involve exploring the combination of MK-0752 with gemcitabine and docetaxel in vivo and in clinical trials for uLMS. 

## Figures and Tables

**Table 1:** Inhibitory concentrations for single agents.

	SK-UT-1B		SK-LMS-1	
Agents	IC_50_ (µM)	IC_30_ (µM)	IC_50_ (µM)	IC_30_ (µM)
MK-0752	4.02 × 10^1^	3.66 × 10^1^	1.35 × 10^2^	8.12 × 10^1^
Docetaxel	5.50 × 10^−5^	2.40 × 10^−4^	1.00 × 10^−2^	8.70 × 10^−3^
Doxorubicin	7.10 × 10^−3^	3.70 × 10^−3^	3.01 × 10^−1^	2.80 × 10^−1^
Gemcitabine	2.00 × 10^−3^	1.40 × 10^−3^	6.00 × 10^−2^	2.00 × 10^−2^

**Table 2:** Inhibitory concentrations for combination agents.

	SK-UT-1B		SK-LMS-1	
Agents	IC_50_ (X)	IC_30_ (µM)	IC_50_ (X)	IC_30_ (µM)
MK-0752 + Docetaxel	-	-	0.80	M: 1.35 × 10^1^, Doce: 1.20 × 10^−3^
MK-0752 + Doxorubicin	0.07	M: 7.30 × 10^−1^, Doxo: 1.00 × 10^−4^	0.80	M: 3.09 × 10^1^, Doxo: 6.90 × 10^−2^
MK-0752 + Gemcitabine	1.05	-	0.30	M: 8.12, Gem: 3.50 × 10^−3^
MK-0752 + Gemcitabine + Docetaxel	0.70	M: 5.04, Gem: 2.50 × 10^−5^, Doce: 6.92 × 10^−5^	0.50	M: 8.03, Gem: 3.30 × 10^−4^, Doce: 6.70 × 10^−4^

M: MK-0752, Doce: docetaxel, Doxo: doxorubicin, Gem: gemcitabine.

**Table 3:** Relative invasion between treatments by cell line.

SK-UT-1B					SK-LMS-1		
Treatment (Ref)	Treatment	Fold Change	*p*-Value	Treatment (Ref)	Treatment	Fold Change	*p*-Value
Doce	MK + Gem + Doce	1.66	0.48	Doce	MK + Doce	1.17	0.25
Doxo	MK + Doxo	1.16	0.39	Doce	MK + Gem + Doce	0.81	0.13
Gem	MK + Gem + Doce	0.75	0.24	Doxo	MK + Doxo	0.82	0.7
				Gem	MK + Gem	1.88	0.24
				Gem	MK + Gem + Doce	0.88	0.58
				MK + Doce	MK + Gem + Doce	0.68	0.13
				MK + Gem	MK + Gem + Doce	0.47	**<0.05**

Ref: reference, M: MK-0752, Doce: docetaxel, Doxo: doxorubicin, Gem: gemcitabine.

**Table 4:** Fold change in Ki-67 expression after treatment relative to DMSO control.

	SK-UT-1B		SK-LMS-1	
Treatment	Fold Change	*p*-Value	Fold Change	*p*-Value
MK-0752	1.09	0.58	0.63	**<0.01**
MK-0752 + Docetaxel	---	---	1.07	0.91
MK-0752 + Doxorubicin	0.99	0.70	1.01	0.94
MK-0752 + Gemcitabine + Docetaxel	1.30	0.83	1.20	0.09

## Data Availability

The RNA sequencing data underlying this article have been uploaded to the Genome Expression Omnibus/National Center for Biotechnology Information with accession number GSE268019. All data presented in this study are available upon request.
